# RNA Interference of Phenoloxidases of the Fall Armyworm, *Spodoptera frugiperda*, Enhance Susceptibility to *Bacillus thuringiensis* Protein Vip3Aa19

**DOI:** 10.3390/insects13111041

**Published:** 2022-11-10

**Authors:** Xiaodan Huang, Dapeng Jing, Sivaprasath Prabu, Tiantao Zhang, Zhenying Wang

**Affiliations:** 1 Engineering Research Center of Natural Enemy Insects, Institute of Biological Control, Jilin Agricultural University, Changchun 130118, China; 2 State Key Laboratory for Biology of Plant Diseases and Insect Pests, Institute of Plant Protection, Chinese Academy of Agricultural Sciences, Beijing 100193, China

**Keywords:** humoral response, enthomopathogens, immune defense, RNAi, PO activity

## Abstract

**Simple Summary:**

Phenoloxidase (PO) is an important part of the innate immune system of the fall armyworm, *Spodoptera frugiperda*. In this study, we screened two PO genes specifically expressed in the hemocyte transcriptome data of the fall armyworm. qRT-PCR results showed that the mRNA expression level of two PO genes gradually increased at different developmental stages. In different tissues, we found that the *Sf*PO2 gene mainly existed in the hemolymph. As an important enzyme for activating the PO gene, the *Sf*PAE gene existed in the midgut and hemolymph. When these two genes were silenced by RNA interference, the pathogenicity of Vip3Aa19 toxin increased significantly. A PO activity assay also confirmed the contribution of two PO genes to insect immune defenses against the Vip3Aa protein.

**Abstract:**

Phenoloxidase (PO) is an important enzyme in the cellular immune system and is involved in defense against a wide range of pathogens, including *Bacillus thuringiensis*. Vip3Aa19 is secreted and expressed by *Bacillus thuringiensis* (Bt) at the middle exponential growth phase and is a kind of protein with efficient insecticidal activity against *Spodoptera frugiperda*. However, immune responses of the target insects have been regarded as a hindrance to Bt pathogenicity. This paper reports two phenoloxidase (PO) genes (*Sf*PAE and *Sf*PO2) identified from the hemocyte transcriptome data of the fall armyworm, *Spodoptera frugiperda.* qRT-PCR validation results showed that the expression levels of two PO genes were significantly upregulated after Vip3Aa19 (LC_50_ = 4.98 µg/g) toxin treatment compared with those of *S. frugiperda* fed an insecticide-free artificial diet. Meanwhile, two PO genes were expressed from the egg to adult stages even without an immune challenge. We noticed that at all developmental stages investigated in the *S. frugiperda*, *Sf*PAE was generally expressed at a higher level than *Sf*PO2. However, after Vip3Aa19 treatment, the *Sf*PO2 gene mRNA expression level was significantly elevated in response to the toxin challenge. An injection of a specific double-stranded RNA (dsRNA) against POs could suppress its expression. The third instar larvae of *S. frugiperda* treated with dsRNA were much more susceptible to Vip3Aa19 toxin than the control larvae were. Notably, the mortality rate was nearly 90% after a dsPO2 injection. These results proved that *Sf*PO2 was more important for the survival of *S. frugiperda*. Finally, RNA interference and then PO activity detection revealed that PO genes mainly existed in the hemolymph and played an important role in immune defense against Bt toxin.

## 1. Introduction

Insects have extremely high species diversity in nature and have formed their innate immune system in their long-term evolution. When exogenous pathogenic microorganisms or the external environment threaten their survival and reproduction, the innate immune system will play a protective role in insects [1]. Insect innate immunity includes cellular immunity and humoral immunity. Cellular immunity involves nodulation, phagocytosis and encapsulation mediated by hemocytes, whereas the humoral immunity is mainly mediated via the activation of prophenoloxidase (PPO) and the induction of antimicrobial peptides (AMPs) [2,3]. Phenoloxidases (POs), also known as tyrosinase or tyrosine hydroxylase, are widely present in animals, plants and microorganisms [4]. PO is expressed in all insects in the form of inactive prophenoloxidase (PPO), which is activated to PO when insects are damaged by environmental disturbances, and this activation process is completed by the prophenoloxidase-activating system (proPO-AS) [5]. With the invasion of exogenous microorganisms, pattern recognition receptors in the proPO-AS in the insect recognize the heterologous substance and bind, cause the cascade reaction, activate the PPO and form PO. After activation, PO oxidizes the phenol in the insect into a quinone and carries out the melanization reaction, triggering melanin synthesis. After melanin deposition, it will engulf and encapsulate pathogens and recruit the hemocytes that can be engulfed [6]. For small particles such as bacteria, the host can eliminate them by phagocytosis, and when the invading foreign microorganism cannot be swallowed by a single blood cell, the host resists annihilation by a melanin-packing reaction. PO is an important enzyme in insects, which plays an important role in metamorphosis and the immune system. The enzyme is mainly involved in three important physiological processes of insects, hardening of insect epidermis, melanization and wound healing of microorganisms [7].

The fall armyworm (FAW), *Spodoptera frugiperda* (J.E. Smith, 1797)*,* has seriously threatened the safety of maize production in China since its invasion in 2019 [8]. Chemical control is the main emergency measure for *S. frugiperda* and results in increasing the use of insecticides [9]. Improper and frequent use of chemical pesticides resulted in the high-level resistance of *S. frugiperda* to several pesticides [10]. Transgenic crops expressing Bt toxins have significantly reduced the use of insecticides, benefited neighboring crops and increased the farmer’s incomes over the past two decades [11,12,13]. *Bacillus thuringiensis* (Bt) is a Gram-positive bacterium with spore formation and exhibits a significant entomopathogenic effect [14]. However, immune responses of the target insects have been regarded as a hindrance to bacterial pathogenicity [15]. Suppression of the host immune system by an immunosuppressant such as pyriproxyfen, a JH analog and benzylideneacetone, also significantly enhances the lethal effect of Bt in some Lepidopteran insects [16,17]. For example, infection of the entomopathogenic fungus *Metarhizium rileyi* suppresses the number of *S. frugiperda* hemocytes and activates humoral immunity of the host *S. frugiperda* [18]. Studies have shown that prophenoloxidases (PPOs) of *S. frugiperda* play a significant role in immune regulation in *Micrococcus luteus* and *Escherichia coli*. Meanwhile, they have been made to identify and characterize these genes in *S. frugiperda* [19]. In Lepidopteran insects, PPO mostly appears in the hemolymph; the Asian corn borer, *Ostrinia furnacalis*, beet armyworm, *Spodoptera exigua*, and silkworm, *Bombyx mori*, have developed resistance to insecticides, and PO activity in the hemolymph was significantly increased [20,21,22]. Studies have found that the activities of PO are significantly increased in Cry1Ac resistance strains of diamondback moth, *Plutella xylostella*, and the higher PO activity may be attributed to the stress of the Cry1Ac toxin [23]. Bt vegetative insecticidal proteins (Vips) are produced by *B. thuringiensis* in the logarithmic growth phase [24]. So far, *S. frugiperda* remains highly sensitive only to Vip3A among many commercially grown Bt maize in the Americas [25]. In this study, Vip3Aa19 toxin was used to treat *S. frugiperda* to explore the relationship between PO genes and Bt protein.

In insects, PO is an important immune system protein. When insects are invaded by foreign objects, humoral and cellular immunity are the main means of defense, and PO plays an important role in this process. Our study found that the phenoloxidase activity of *S.frugiperda* increased significantly after feeding on an artificial diet containing Vip3Aa19 toxin, and the higher PO activity may be attributed to the stress of Vip3Aa toxin. Thus, we want to know what the responses of phenoloxidase and its related genes are, and whether that response is related to the Bt insecticides resistance. This study identified two differentially expressed PO genes, *Sf*PAE (*S. frugiperda* phenoloxidase-activating enzyme-like) and *Sf*PO2 (*S. frugiperda* phenoloxidase subunit 2-like) expressed in hemocyte transcriptome data of *S. frugiperda* treated with Vip3Aa19 toxin. Using RNA interference (RNAi), the significance of POs in defending the insect from a Vip3Aa19 toxin infection was assessed. Meanwhile, a study about the activity of PO enzyme after an infection with Vip3Aa19 toxin was conducted. The present study aims to analyze the alteration of PO gene expression and PO activity in *S. frugiperda* in response to Vip3Aa19 toxin on different tissues and larval carcass tissues. This study offers insights into the physiological roles of PO genes in the immune system of *S. frugiperda*.

## 2. Materials and Methods

### 2.1. Insects

*S. frugiperda* larvae were obtained from a laboratory colony at the Institute of Plant Protection of the Chinese Academy of Agricultural Sciences (IPP, CAAS). The original larvae were collected from cornfields in Mangshi, Dehong Prefecture, Yunnan Province, China, and cultured continuously on an insecticide-free artificial diet with 65 ± 5% RH, 26.5 ± 0.5 °C and 14 h L:10 h D.

### 2.2. Bt Toxin and Bioassays

The Vip3Aa19 toxin was provided by Beijing DaBeiNong Biotechnology Co., Ltd. (Beijing, China). Vip3Aa19 toxin was used to check the toxicity against *S. frugiperda* neonates and third instar larvae. Initially, the Vip3Aa19 toxin stock solution (1 mg/mL) was prepared by dissolving the toxin in a sodium carbonate buffer (50 mM, pH = 10). The Vip3Aa19 toxin solution was diluted with distilled water to eight concentration gradients (0.0 µg/g, 0.2 µg/g, 0.5 µg/g, 1.0 µg/g, 2.5 µg/g, 5.0 µg/g, 10.0 µg/g and 25.0 µg/g) before mixing into an artificial diet. The calculation method of protein addition is a gradient concentration of protein (µg/g) multiplied by 10 g then divide by 1 mg/mL. Each 24-well plate contained 9 g of the artificial diet and Vip3Aa19 toxin diluted to a total of 1 mL. When the agar artificial diet was cooled below 42 °C, 1 mL of the Vip3Aa19 toxin diluent was added and stirred well. The cut and mixed artificial diet was placed in a 24-well cell culture plate, ensuring that each well contained the same amount of the artificial diet. One neonate or third instar larva was placed on the surface of the diet in each well using a fine brush. These plates were sealed with sealing film, a small hole was punctured on each well, and then placed in the rearing room (65 ± 5% RH, 26.5 ± 0.5 °C and 14 h L:10 h D). Each concentration was replicated in three plates and contained a total of 72 larvae. Neonatal mortality was checked after seven days. Third instar larvae mortality was estimated after 10 days. Because the third instar larvae are well-developed, they already have fine immune ability, needing more time for observation. If larvae died or could not move coordinately, they were considered dead. The number of larval deaths and survivals in three 24-well plates at each concentration gradient was recorded statistically. The data were processed with Polo Plus.

### 2.3. Sample Preparation

Third instar larvae of *S. frugiperda* were treated with an LC_50_ (4.98 µg/g) dose of Vip3Aa19 for 48 h with the same method as described in Section 2.2 before the sample collection, while the control group was fed under the same conditions with an insecticide-free agar artificial diet. Each 24-well plate contained 9 g of the artificial diet with 1 mL of distilled water and was stirred well. The test conditions in both the control group and the treated group were conducted in the same way. Each treatment and control group comprised three replications with 24 third instar larvae per replicate. A hemocyte was extracted for the determination of the hemocyte transcriptome. All samples were stored at −80 °C for further analyses. RNA-seq analyses were performed by Biomarker Technologies Co., Ltd. (Beijing, China).

### 2.4. RNA Extraction and cDNA Synthesis

Total RNA samples were extracted using each developmental stage of *S. frugiperda*. Meanwhile, 24 third instar larvae were used to extract the midgut, hemocyte and fat body.

Total RNA was extracted from each sample with the TRIzol method. The quality of the total RNA from individual tissue samples was evaluated with electrophoresis in 1% agarose gels, and RNA was quantified spectrophotometrically with a NanoDrop 2000 spectrophotometer (Thermo Scientific, Waltham, DE, USA) and an Agilent Bioanalyzer 2100 (Agilent, Santa Clara, CA, USA). All RNA samples were stored at −80 °C. First-strand cDNA was synthesized using an RT™ All-in-One Master Mix Kit (Herogen Biotech, Shanghai, China). cDNA synthesis was carried out using 1 μg of total RNA. Anchored oligo (dT) was used to synthesize cDNA. The final cDNA samples were stored at −20 °C until further analysis.

### 2.5. Quantitative Real-Time PCR (qRT-PCR)

The expression of *Sf*PAE and *Sf*PO2 genes in each developmental stage and in different tissues of *S. frugiperda* were analyzed using qRT-PCR. The SYBR green (Premix ExTaq^TM,^ Takara, Japan) method was used to evaluate the expression level of two genes in different tissues of *S. frugiperda* (ABI 7500 Fast, Applied Biosystems, Waltham, MA, USA). qRT-PCR primer pairs were designed using Primer 3 (http://bioinfo.ut.ee/primer3/ (accessed on 13 October 2022)) (Table 1). A two-step program was adopted; reaction volume was set to 20 µL. The program was designed as follows: denaturation at 95 °C for 30 s, followed by 40 cycles of 95 °C for 5 s, 60 °C for 30 s; melting curve analysis was performed from 60 to 95 °C to determine the specificity of the PCR products. Three independent biological replicates were maintained for all the samples, and three technical replicates were performed from each biological sample. The 2^−∆∆CT^ method was used to calculate the expression of PO genes relative to the reference gene GADPH [26,27].

### 2.6. RNA Interference (RNAi)

The third instar larvae of *S. frugiperda* were utilized to examine the role of two PO genes against Bt toxicity by RNAi. The *Sf*PAE and *Sf*PO2 primers were designed to synthesize dsRNA. Meanwhile, specific primers dsGFP-F and dsGFP-R were designed according to a GFP sequence as control (Table 1). Double-stranded RNA (dsRNA) was synthesized according to a T7 Ribo MaxTM Express RNAi System Kit (Promega, Beijing Biotech Co., Ltd, Beijing, China), and the final dsRNA concentration was adjusted to 2500 ng/μL. Into the abdomen of the third instar larvae of *S. frugiperda*, 100 nL of dsRNA was injected using a glass capillary injection needle, which was made using a Micropipette puller PN-30 (Narishige, Tokyo, Japan). To restrict insect movement, they were cooled for two minutes before the injection. Control groups were either injected with equivalent volumes of ddH_2_O. Meanwhile, a similar dsRNA construct of the green fluorescence protein gene, dsGFP, was generated as a control. RNA was extracted from whole third instar larvae at different time points (24, 48, and 72 h) after feeding an artificial diet, and qRT-PCR was used to detect the gene interference efficiency. The procedures were performed as above.

Third instar larvae of *S. frugiperda* were treated with dsRNA specific to POs as described above. The results of qRT-PCR showed that the expression of PO genes decreased significantly after 48 h of injection of dsRNA. Therefore, 48 h was selected to feed the artificial diet of the 3rd instar larvae of *S.frugiperda* and mixed with Vip3Aa19 toxin. The procedures were performed the same as in Section 2.2. Four treatments (injection ddH_2_O; injection dsGFP; injection dsPAE; injection dsPO2) were replicated four times, and each replication used 24 larvae and contained a total of 96 larvae. After 48 h, the 72 fine surviving larvae were selected and fed an artificial diet supplemented with Vip3Aa19 toxin. Larvae mortality and the weights of surviving larvae were estimated after 10 days.

### 2.7. Midgut, Hemolymph and Hemocyte Sample Preparation and PO Activity Assay

PO activity was analyzed in the midgut, hemolymph and hemocytes lysate supernatant (HLS). The midgut of the third instar larva of *S. frugiperda* was dissected and washed using an ice-cold Mannitol buffer (300 mM Mannitol, 17 mM Tris-HCl, 5 mM EGTA, 2 mM DDT and 0.5 mM PMSF, pH = 7.4). The midgut tissue was transferred to a glass homogenizer and homogenized using an ice-cold lysis buffer (50 mM Tris-HCl, 150 mM NaCl, 1% NP-40, 0.25% Na-deoxycholate, 1 mM Protease Inhibitor Cocktail (Roche, Mannheim, Germany, pH = 7.4). The homogenate was transferred to a microfuge tube, placed on ice for 10 min and centrifuged (10,000× *g* for 15 min at 4 °C). The supernatant was recovered, and the total protein extracted from the midgut was used to assess PO activity. The hemolymph was extracted by cutting the proleg of the larvae, and oozing hemolymph was collected in a sterile microfuge tube held on ice and centrifuged (800× *g*, 15 min, 4 °C). The hemocyte-free supernatant was used to assess PO activity. The HLS was prepared by cutting the proleg of the larva, and oozing hemolymph was collected in a microfuge tube containing 500 μL of ice-cold iso-osmotic buffer (50 mM Tris-HCl, 10 mM CaCl_2_, 100 mM NaCl, 100 mM dextrose, 6 mM KCl, 5 mM MgCl_2_, pH = 7.2, 300 mOsm with cOmplete tablet EASYpack Protease Inhibitor Cocktail, Roche, GmbH, Germany) held on ice. This hemocyte suspension was centrifuged (400× *g*, 5 min, 4 °C), and the supernatant was discarded. The loosely packed hemocyte pellet was washed twice by centrifuge (400× *g*, 5 min, 4 °C) using the iso-osmotic buffer. Then, the iso-osmotic buffer was discarded, and the pellet was homogenized using 100 μL of ice-cold lysis buffer. The homogenate was centrifuged (10,000× *g* for 15 min at 4 °C), and the supernatant (HLS) was used to assess PO activity. The protein concentrations in the samples were estimated using an Easy Protein quantitative kit (TransGen Biotech Co., Ltd., Beijing, China), and the sample concentrations were set to 1 mg/mL using Tris-buffered saline (50 mM Tris-HCl, 10 mM CaCl_2_, 100 mM NaCl, pH = 7.2 with cOmplete tablet EASYpack Protease Inhibitor Cocktail, Roche, GmbH, Mannheim, Germany) before subjecting to PO activity.

In vitro PO activity in the midgut and hemolymph fractions (serum and HLS) was assessed in the non-treated control, Vip3Aa19 toxin treatment, only RNAi treatment and both RNAi- and Vip3Aa19-toxin-treated samples. PO activity was determined by incubating the samples prepared from the midgut and hemolymph with 5 mM L-3,4-dihydroxyphenylalanine (L-DOPA, MedChemExpress, Monmouth Junction, NJ, USA) in Tris-HCl buffer (250 mM, pH = 6.5, freshly prepared before use, with cOmplete tablet EASYpack Protease Inhibitor Cocktail, Roche, GmbH, Germany). This was done by incubating 20 μL of the midgut or serum or HLS samples with 180 μL of L-DOPA in a 96-well plate for 10 min at RT. The formation of dopachrome was measured at 480 nm in a spectrophotometer (FlexStation 3, Molecular Devices, CA, USA) against a blank, and six replicates were maintained for all prepared samples. PO activity was expressed as units min^−1^ mg protein^−1^ [24]. One unit of enzyme activity is defined as the amount of enzyme that in 10 min gives an increase of 0.001 in absorbency at 480 nm.

### 2.8. Statistical Analysis

The LC_50_ value for Vip3Aa19 toxin was calculated using Polo Plus software (v1.0, LeOra Software, Parma, MO, USA). Data related to all experiments and the relative mRNA expression levels of the PO genes were analyzed using the SPSS 20.0 Software Package, and Tukey’s test was used to determine the variance of the significance level (*p* < 0.05) (SPSS Inc., Chicago, IL, United States). All figures were plotted by GraphPad Prism 5.0 software (GraphPad, San Dieg, CA, USA).

## 3. Results

### 3.1. Differential PO Genes Screening and qRT-PCR Verification

**Figure 1:**
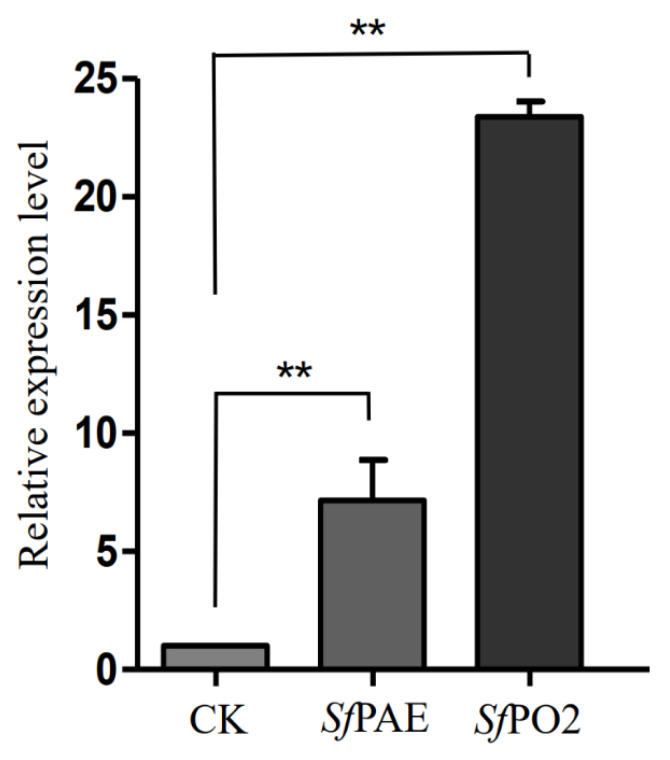
qRT-PCR measurement of *Sf*PAE and *Sf*PO2 mRNA expression levels treated with Vip3Aa19 toxin of *S. frugiperda*. CK: control group. Vertical bars under the asterisk indicate significant differences based on Tukey’s test (** *p-*value < 0.01).

Two differentially expressed PO genes were screened from the hemocyte transcriptome data of *S. frugiperda* treated with Vip3Aa19 toxin, which were *Sf*PAE (*S. frugiperda* phenoloxidase-activating enzyme-like) and *Sf*PO2 (*S. frugiperda* phenoloxidase subunit 2-like). *S. frugiperda* is sensitive to Vip3Aa19 toxin. The LC_50_ of Vip3Aa19 toxin to third instar larvae was 4.98 µg/g (Table S1). The hemocyte total RNA was extracted after 48 h of treatment, and the sensitive larvae fed with a normal artificial diet without any toxin exposure were taken as the control group. The qRT-PCR results showed that the expression levels of two PO genes were significantly upregulated when treated with Vip3Aa19 after 48 h. The expression of the *Sf*PO2 gene was significantly higher than that of the *Sf*PAE gene (Figure 1).

### 3.2. S. frugiperda POs Expression Profiles

**Figure 2:**
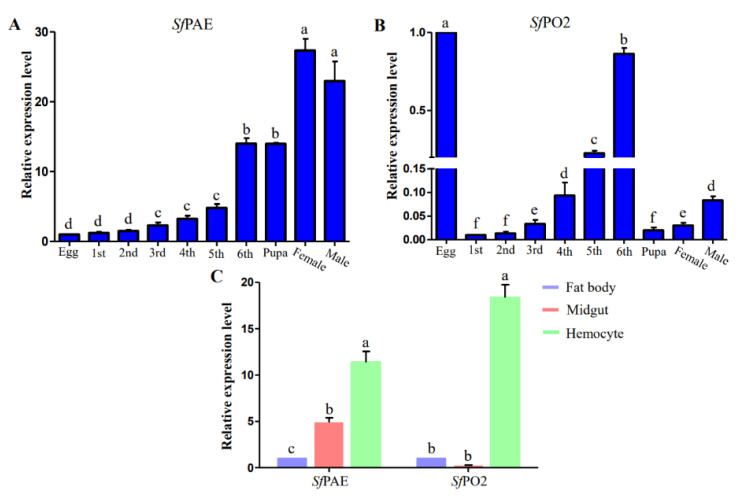
qRT-PCR measurement of *Sf*PAE and *Sf*PO2 expression at different time points during the life cycle of *S. frugiperda.* (**A**) *Sf*PAE gene, (**B**) *Sf*PO2 gene. qRT-PCR measurement of *Sf*PAE and *Sf*PO2 expression at different tissues (**C**). Bars with the same letter are not significant, and different letters indicate a significant difference from each other at *p* < 0.05 based on Tukey’s test.

The study verified that these annotated genes were transcribed in *S. frugiperda* caterpillars by performing a series of qRT-PCR for two POs at all different developmental stages and in different tissues. We noticed that at all investigated stages, *Sf*PAE was generally expressed at higher levels than *Sf*PO2 was (Figure 2). *Sf*PAE was detected in eggs and larvae, and its expression level gradually increased, with maximum expression occurring in adults, and expression level significantly different from that in other developmental periods (Figure 2A). The expression level of the *Sf*PO2 gene in the whole insect was generally low (Figure 2B). However, the mRNA expression level in old instar larvae was significantly higher than that in young instar larvae. Meanwhile, the expression levels of male and female adults were also significantly different. We also investigated the tissue-specific expression of two PO genes for *S. frugiperda*. Two PO genes were highly expressed in hemocytes, significantly higher than in the midgut and fat body. In the midgut and hemocyte, the mRNA expression of *Sf*PAE gene was significantly different, but there was no difference in the *Sf*PO2 gene (Figure 2C).

### 3.3. RNAi-Mediated Downregulation of dsPAE and dsPO2 Genes in S. frugiperda via qRT-PCR

**Figure 3:**
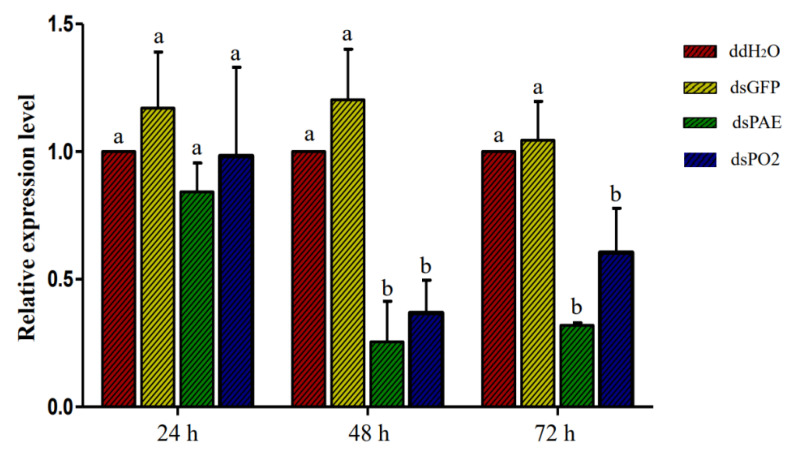
The relative mRNA transcript levels of *S. frugiperda* larvae after injecting with ddH_2_O, dsGFP, dsPAE and dsPO2 for 24, 48 and 72 h. Bars with the same letter are not significant, and different letters indicate a significant difference from each other at *p* < 0.05 based on Tukey’s test.

**Figure 4:**
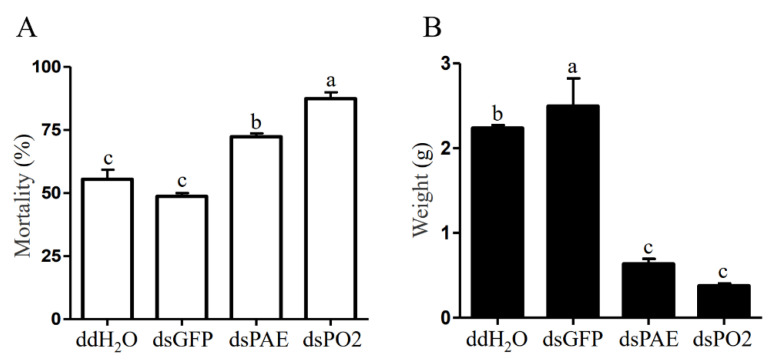
Comparison of mortality and weight of surviving larvae injected with different dsRNAs. (**A**) Mortality of larvae, (**B**) weight of surviving larvae. Bars with the same letter are not significant, and different letters indicate a significant difference from each other at *p* < 0.05 based on Tukey’s test.

Two target PO genes were selected to evaluate their function in Bt insecticidal activity of *S. frugiperda*. qRT-PCR was conducted to check the relative change in mRNA expression of targeted genes in *S. frugiperda* larvae injected with dsRNAs, dsPAE and dsPO2 along with the ddH_2_O and dsGFP control for 24, 48 and 72 h. Dramatically lower mRNA transcript levels of dsPAE and dsPO2 genes were observed after 48 h and 72 h compared with those of ddH_2_O and dsGFP. There was no difference between dsGFP and ddH_2_O. After 24 h of treatment, the gene expression levels of *Sf*PAE and *Sf*PO2 showed no significant downward trend, while the expression levels at 48 h and 72 h were significantly downregulated (Figure 3). Therefore, we selected 48 h of dsRNA injection, and *S. frugiperda* was fed an artificial diet mixed with Vip3Aa19. Bioassay results showed no significant difference in mortality between GFP and ddH_2_O injection after 10 days, close to 50%. After injection of dsPAE and dsPO2 and feeding, the mortality increased significantly, to more than 70%, and the mortality of *Sf*PO2 gene silencing was significantly higher than that of *Sf*PAE gene silencing (Figure 4A). In addition to calculating mortality, we also compared the differences between the weights of the surviving larvae. The results showed that after silencing the two PO genes in insects, although dsRNA only played a role for a period of time, it seriously affected the growth of *S. frugiperda*. Compared with that in the control treatment group, the weight of the surviving larvae of *S. frugiperda* decreased, with a significant difference (Figure 4B).

### 3.4. PO Activity in the Midgut, Hemolymph and Hemocyte Lysate Supernatant

**Figure 5:**
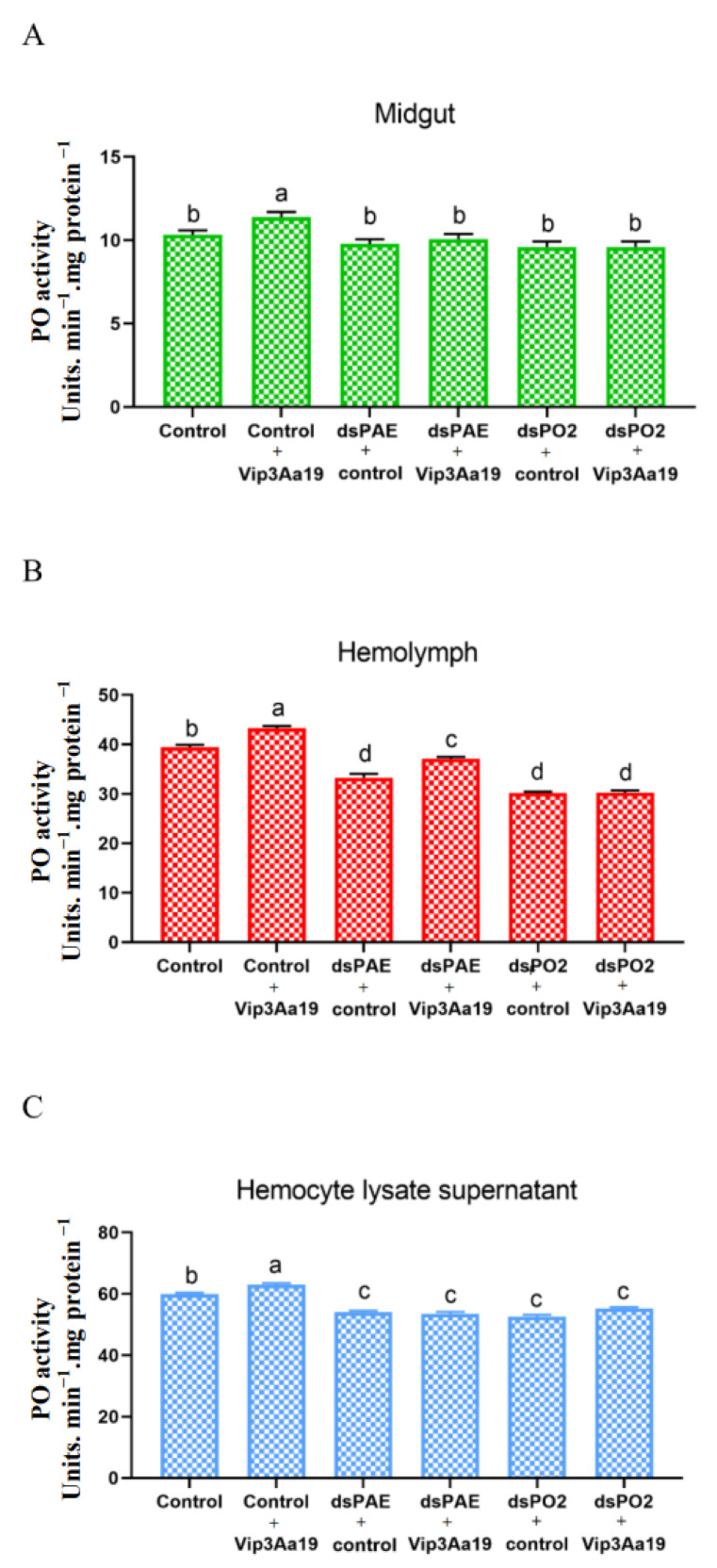
PO activity assessment in the midgut, hemolymph and hemocyte lysate supernatant samples of *S. frugiperda.* (**A**). PO activity in midgut samples extracted from control and Vip3Aa19 toxin treatment in *Sf*PAE and *Sf*PO2 knockdown larvae. (**B**). PO levels in hemocyte-free hemolymph samples collected from control and Vip3Aa19 toxin treatment in *Sf*PAE and *Sf*PO2 knockdown larvae. (**C**). PO activity in hemocyte lysate supernatant samples collected from control and Vip3Aa19 toxin treatment in *Sf*PAE and *Sf*PO2 knockdown larvae. PO activity was based on six replicates (mean ± SEM). Vertical bars under different letters within the sample tested were statistically significant (Tukey’s, *p* < 0.05), and the bars with the same letter are insignificant.

PO activity was assessed in the midgut, hemolymph and hemocyte lysate supernatant samples of control and RNAi knockdown larvae. The larvae without dsRNA treatment but treated with Vip3Aa19 toxin showed a significant increase in PO activity in all three samples tested (Figure 5). After gene silencing of SfPAE and SfPO2, the larvae were fed an artificial diet supplemented with Vip3Aa19 toxin. Compared with the larvae without toxin treatment, PO activity in the midgut did not change significantly. Compared with the control + Vip3Aa19, PO activity was significantly reduced with RNAi knockdown + Vip3Aa19 (Figure 5A). The dsPAE + Vip3Aa19-treated hemolymph samples showed significantly higher PO activity than the other dsRNA-treated larvae did (Figure 5B). Similarly, the hemocyte lysate supernatant of dsRNA-treated larvae showed a significant decrease in PO activity (Figure 5C).

## 4. Discussion

PO plays an important role in the growth and development of insects and is a key enzyme for melanin synthesis and insect immune processes, and PO activity is regarded as an important marker of host immunity [28,29]. *B. thuringiensis* (Bt), the most widely used and successful microbial insecticide, has specific high insecticidal activity against field pests [30]. In America, with the continuous cultivation of Bt crops, *S. frugiperda* has developed different degrees of resistance to insect-resistant maize expressing Cry1Ab and Cry1F proteins [31]. Recent research demonstrates that the members of the Vip3A toxin family show high insecticidal activity against a wide range of Lepidopteran pests [32]. Our study also showed that Vip3Aa19 toxin had high toxicity to neonate and third instar larvae of *S. frugiperda*. Meanwhile, the sensitivity of *S. frugiperda* to Bt toxin is closely related to the expression of immune gene POs and PO enzyme activity.

In this study, the sensitive *S. frugiperda* was used as the research material, and Vip3Aa19 was used to infect *S. frugiperda* to explore its innate immune system, especially the response of PO genes to Bt when infected with Vip3Aa19 toxin. Specific upregulated *Sf*PAE (*S. frugiperda* phenoloxidase-activating enzyme-like) and *Sf*PO2 (*S. frugiperda* phenoloxidase subunit 2-like) were screened by hemocyte transcriptome data of *S. frugiperda* treated with Vip3Aa19 toxin. qRT-PCR results revealed that the mRNA expression levels of the two PO genes in the hemocyte were significantly upregulated after treatment with Vip3Aa19 toxin compared to those in the untreated group. Recent research demonstrates that PO activity and mRNA transcriptional expression and protein levels of the PPO gene in resistant strains are all significantly higher than in the susceptible strain of *P. xylostella* [33]. PO usually exists as an inactive prophenoloxidase (PPO) that is stored in the hemolymph. The activation process culminates in the activation of prophenoloxidase (PPO), which drives the production of melanin. Therefore, while PPO affects the immune function of PO, its expression and activity also affect the immune function of the host. More importantly, the expression of *Sf*PO2 was higher than that of *Sf*PAE. Both PO genes are specifically upregulated upon Vip3Aa19 toxin invasion. The innate immune system response is central for insects to sequester invading pathogens. POs were already identified as indispensable for the *S. frugiperda* innate immunity in both cellular and humoral aspects [34]. This study also showed that *Sf*PAE and *Sf*PO2 are expressed in all developmental stages and tissues of *S. frugiperda*. *Sf*PAE gene expression levels upregulated with increasing age, and the mRNA expression level reached the maximum in the adult stage. It indicated that *Sf*PAE possibly not only functioned as one of the key factors to compete with defense stress in larvae, but also was endowed with the physical function in adults’ development and may be involved in the melanization and hardening of adult body wall and its extremity [35]. This result agreed well with other research that found a significant role of adult *Sf*POs in the growth and development of *S. frugiperda* [36]. The overall expression level of *Sf*PO2 was lower than that of *Sf*PAE. This may be because *Sf*PO2 mainly exists in the hemolymph of insects and is less expressed in other tissues [33]. *Sf*PAE is an important activating enzyme in proPO-AS. We speculate that *Sf*PAE can activate a variety of POs genes to play an immune function of the host, so *Sf*PAE may exist in both the hemocyte and hemolymph. With detection of PO gene expression in different tissues, we also found that *Sf*PAE was mainly present in hemocytes in sensitive *S. frugiperda* populations, but it was also significantly expressed in the midgut compared to the fat body. *Sf*PO2 mainly exists in the hemocyte with a small amount in the midgut. In *Drosophila*, PO mutants show increased susceptibility to pathogens [37]. In addition, POs have also been shown to play a role in wound healing and clotting [38,39], suggesting additional PO roles beyond immunity. In addition to contributing to the immune enhancement of pests, PO also has side effects on beneficial insects through immunosuppression. For example, studies have found that honey bees treated with different doses of neonicotinoid insecticides at different times had reduced activities of proPOx and POx in the hemolymph [40]. Additionally, the herbicide pendimethalin was found to affect the level of plasmatic PO and lysozyme-like enzyme activities of ground beetle, thus modifying the immunocompetence of the beneficial ground beetle [41].

This study used RNA interference to silence *Sf*PAE and *Sf*PO2 genes in *S. frugiperda*. dsPAE and dsPO2 genes were injected and fed LC_50_ Vip3Aa19 toxin. It is noteworthy that the mortality of larvae was close to 90% after the dsPO2 injection. Meanwhile, silencing both PO genes made it difficult for *S. frugiperda* to gain weight over time. We boldly speculate that insects have difficulties successfully developing into adults and laying eggs. Four PPO genes have been reported in *S. frugiperda* [19], and dsPO2 is an important immune enzyme in the immune system. Our study may also prove that *Sf*PO2 is a more important immunity gene in *S. frugiperda* with a specific immune function to Bt toxin. One finding also implies that suppressing immune responses may increase the gut region’s Bt toxicity and the possibility of the gut microbiome adapting to commensal pathogens in the hemocoel [42]. In our study we observed that the growth of the third instar larvae of *S. frugiperda* was significantly inhibited and they failed to molt, leading to sepsis and death. It could be speculated that the immune responses of PO may be the natural response of resistance.

In order to verify the POs activity of *S. frugiperda* in the midgut, hemolymph and hemocytes after feeding Vip3Aa19 toxin and silencing the PO genes, we determined PO content in *S. frugiperda* fed on insecticidal protein Vip3Aa19 and silenced *Sf*PAE and *Sf*PO2 genes by an enzyme activity assay. The activity of PO in the midgut, hemolymph and hemocyte lysate supernatant increased significantly after feeding the Vip3Aa19 toxin compared with those in the control. Consistently with qRT-PCR results, the POs mRNA expression level was upregulated. As a result, we conclude that the increased PO activity in the tested tissues could be a response to Vip3Aa19 toxicity. It is known that insect PPO not only plays an important role in shielding hosts from invasion (immediate activation in vivo) but is also responsible for wound healing [37,43,44]. Therefore, PPOs may also exist in small amounts in other tissues. In the hemolymph group, when the dsPAE gene was silenced, the larvae were immediately treated with Vip3Aa19 toxin, and PO activity was found to be significantly higher than that of untreated larvae. This indicated that *Sf*PAE activity decreased after silencing, but after Vip3Aa19 toxin, *Sf*PAE exercised a significant activation, so that more PPO was activated to PO to play an immunity function. Therefore, it is further confirmed that *Sf*PAE also exists in the hemolymph and is positively correlated with PO. As a specific enzyme catalyzing PPO, PAE also plays an important role in the immune system [45]. In the hemocyte lysate supernatant, after silencing and Vip3Aa19 toxin treatment, there was no significant difference compared with the control. This may be due to the fact that PO cleaves into the hemolymph after hemocyte disruption, resulting in decreased PO activity in hemocytes. However, more research is needed to determine the molecular mechanism underlying the increase in PO activity. Research finds that the knockdown of PPO by gene silencing in third instar larvae of *Zeugodacus tau* induces abnormal puparia and low pupation rates, which is important for the normal transition from larvae to pupae [46]. Similarly, increased PO activity may be induced by the selective pressure of the Bt protein. It has been demonstrated that host-plant effects can partially suppress PO activity in *P. xylostella* [47].

In summary, innate immunity is an important part of insect defense against external damage. The PO gene is an important group in humoral and cellular immunity, but there are few studies on the pathogenicity of Bt to the fall armyworm. This study showed that PO genes are immune genes against Gram-positive bacteria and play dual roles in development and immune responses. The activity of PO increased significantly after feeding with Vip3Aa toxin. Similarly, the suppression of POs expression enhanced the pathogenicity of Vip3Aa19 toxin. POs, as a resistance factor against Bt, need to be tested for directly exhibiting the adverse effect on the bacteria or indirectly giving an antibacterial effect on indigenous gut bacteria to suppress septicemia due to the effect of Bt toxins.

## 5. Conclusions

In this study, the response of the innate immune system of *S. frugiperda*, especially proPO-AS, to Vip3Aa19 toxin, was preliminarily analyzed by transcriptome sequencing and gene interference. Our results revealed that two PO genes were expressed in all development stages of *S. frugiperda* and increased with age. After silencing *Sf*PAE and *Sf*PO2 genes, the bioassay and POs activity was measured to verify the important role of two PO genes in innate immunity. In general, the results provide insights into the physiological roles of PO genes in the immune system of *S. frugiperda*.

## Figures and Tables

**Table 1:** Primer information.

Gene Name	Genes	Primer	Sequences (5′3′)	Amplified Fragment Length (bp)
*Sf*PAE	LOC118279360	qPAE-F	CGGCCCATCTGTTTACCAAC	168 bp
		qPAE-R	GTACGCTGGTTCGCATTGAT	
dsPAE		dsPAE-F	GGATCCTAATACGACTCACTATAGGATTAGGCGTTGGACTGGTG	433 bp
		dsPAE-R	GGATCCTAATACGACTCACTATAGGCTCATCGCGCGTTCTTGAAT	
*Sf*PO2	LOC118275234	qPO2-F	CATACACAACAACGGGCACA	170 bp
		qPO2-R	ACTGAGACTCCTTGTGCCTC	
dsPO2		dsPO2-F	GGATCCTAATACGACTCACTATAGGTGGTCCCAATTCTCGTGGAAC	452 bp
		dsPO2-R	GGATCCTAATACGACTCACTATAGGCTTGAAGTTGACCTTGATGCC	
GADPH	AF400219.1	GADPH-F	CGGTGTCTTCACAACCACAG	209 bp
		GADPH-R	TTGACACCAACGACGAACAT	
dsGFP	AB062168.1	dsGFP-F	GGATCCTAATACGACTCACTATAGGGTGGTCCCAATTCTCGTGGAAC	468 bp
		dsGFP-F	GGATCCTAATACGACTCACTATAGGGCTTGAAGTTGACCTTGATGCC	

## Data Availability

The data presented in this study are available on request from the corresponding author. The data are not publicly available due to restriction eg privacy.

## Supplementary Materials

The following supporting information can be downloaded at: https://www.mdpi.com/article/10.3390/insects13111041/s1, Table S1: Susceptibility of *S. frugiperda* neonates and third instar larvae to Vip3Aa19 toxin.

## References

[1] Lemaitre B., Hoffmann J. (2007). The Host Defense of *Drosophila melanogaster*. Annu. Rev. Immunol..

[2] Strand M.R. (2008). The insect cellular immune response. Insect Sci..

[3] Rosales C. (2011). Phagocytosis, a cellular immune response in insects. Invert. Surv. J..

[4] Kim M.S., Baek M.J., Lee M.H., Park J.W., Lee S.Y., Söderhäll K., Lee B.L. (2002). A New Easter-type Serine Protease Cleaves a Masquerade-like Protein during Prophenoloxidase Activation in Holotrichia diomphalia Larvae. J. Biol. Chem..

[5] Lu A., Zhang Q., Zhang J., Yang B., Wu K., Xie W., Luan Y.-X., Ling E. (2014). Insect prophenoloxidase: The view beyond immunity. Front. Physiol..

[6] Söderhäll K., Cerenius L. (1998). Role of the prophenoloxidase-activating system in invertebrate immunity. Curr. Opin. Immunol..

[7] Andersen S.O., Peter M.G., Roepstorff P. (1996). Cuticlar sclerotization in insects. Comp. Biochem. Physiol. Physiol..

[8] Jing D.P., Guo J.F., Jiang Y.Y., Zhao J.Z., Sethi A., He K.L., Wang Z.Y. (2020). Initial detections and spread of invasive Spodoptera frugiperda in China and comparisons with other noctuid larvae in corn fields using molecular techniques. Insect Sci..

[9] Yang X., Wyckhuys K.A., Jia X., Nie F., Wu K. (2021). Fall armyworm invasion heightens pesticide expenditure among Chinese smallholder farmers. J. Environ. Manag..

[10] Gui F., Lan T., Zhao Y., Guo W., Dong Y., Fang D., Liu H., Li H., Wang H., Hao R. (2020). Genomic and transcriptomic analysis unveils population evolution and development of pesticide resistance in fall armyworm Spodoptera frugiperda. Protein Cell.

[11] Shelton A.M., Zhao J.Z., Roush R.T. (2002). Economic, Ecological, Food Safety, and Social Consequences of the Deployment of Bt Transgenic Plants. Annu. Rev. Èntomol..

[12] Tabashnik B.E., Sisterson M.S., Ellsworth P.C., Dennehy T.J., Antilla L., Liesner L., Whitlow M., Staten R.T., Fabrick J.A., Unnithan G.C. (2010). Suppressing resistance to Bt cotton with sterile insect releases. Nat. Biotechnol..

[13] Chandrasena D.I., Signorini A.M., Abratti G., Storer N.P., Olaciregui M.L., Alves A.P., Pilcher C.D. (2018). Characterization of field-evolved resistance to Bacillus thuringiensis-derived Cry1F δ-endotoxin in Spodoptera frugiperda populations from Argentina. Pest Manag. Sci..

[14] Lu Y., Wu K., Jiang Y., Guo Y., Desneux N. (2012). Widespread adoption of Bt cotton and insecticide decrease promotes biocontrol services. Nature.

[15] Rahman M., Roberts H., Sarjan M., Asgari S. (2004). Induction and transmission of Bacillus thuringiensis tolerance in the flour moth, Ephestia kuehniella. Proc. Natl. Acad. Sci. USA.

[16] Kwon S., Kim Y. (2007). Immunosuppressive action of pyriproxyfen, a juvenile hormone analog, enhances pathogenicity of Bacillus thuringiensis subsp. kurstaki against diamondback moth, Plutella xylostella (Lepidoptera: Yponomeutidae). Biol. Control.

[17] Kwon B., Kim Y. (2008). Benzylideneacetone, an immunosuppressant, enhances virulence of Bacillus thuringiensis against beet armyworm (Lepidoptera: Noctuidae). J. Econ. Entomol..

[18] Wang J., Yang K., Wang S., Li X., Liu J., Yu Y., Liu X. (2022). Infection of the entomopathogenic fungus *Metarhizium rileyi* suppresses cellular immunity and activates humoral antibacterial immunity of the host *Spodoptera frugiperda*. Pest Manag. Sci..

[19] Yang L., Xing B., Wang L., Yuan L., Manzoor M., Li F., Wu S. (2021). Identification of serine protease, serine protease homolog and prophenoloxidase genes in Spodoptera frugiperda (Lepidoptera: Noctuidae). J. Asia-Pac. Èntomol..

[20] Prabu S., Jing D., Shabbir M.Z., Yuan W., Wang Z., He K. (2020). Contribution of phenoloxidase activation mechanism to Bt insecticidal protein resistance in Asian corn borer. Int. J. Biol. Macromol..

[21] Wang X., Liu C., Zhang J.-D., Luo W.-C. (2005). Inhibitory kinetics of quercetin on phenoloxidase from loopworm. Insect Sci..

[22] Tang F.F., Yang W.K., Zhu F., Shao Y.L., Zhang Y.H., Bai X.R. (2016). BmNPV affecting the activity and gene expression of phenoloxidase in Bombyx mori. Chin. Agricul. Sci. Bull..

[23] Liu A., Huang X., Gong L., Guo Z., Zhang Y., Yang Z. (2019). Characterization of immune-related PGRP gene expression and phenoloxidase activity in Cry1Ac-susceptible and -resistant *Plutella xylostella* (L.). Pestic. Biochem. Physiol..

[24] Estruch J.J., Warren G.W., Mullins M.A., Nye G.J., Craig J.A., Koziel M.G. (1996). Vip3A, a novel Bacillus thuringiensis vegetative insecticidal protein with a wide spectrum of activities against lepidopteran insects. Proc. Natl. Acad. Sci. USA.

[25] Fatoretto J.C., Michel A.P., Silva-Filho M., Silva N. (2017). Adaptive Potential of Fall Armyworm (Lepidoptera: Noctuidae) Limits Bt Trait Durability in Brazil. J. Integr. Pest Manag..

[26] Livak K.J., Schmittgen T.D. (2001). Analysis of relative gene expression data using real-time quantitative PCR and the 2 (-Delta Delta C(T)) method. Methods.

[27] Shu B.-S., Yu H.-K., Dai J.-H., Xie Z.-G., Qian W.-Q., Lin J.-T. (2021). Stability evaluation of reference genes for real-time quantitative PCR normalization in Spodoptera frugiperda (Lepidoptera: Noctuidae). J. Integr. Agric..

[28] Cerenius L., Söderhäll K. (2004). The prophenoloxidase-activating system in invertebrates. Immunol. Rev..

[29] Nappi A., Christensen B. (2005). Melanogenesis and associated cytotoxic reactions: Applications to insect innate immunity. Insect Biochem. Mol. Biol..

[30] Sanahuja G., Banakar R., Twyman R.M., Capell T., Christou P. (2011). Bacillus thuringiensis: A century of research, development and commercial applications. Plant Biotechnol. J..

[31] He K.L., Wang Z.Y. (2020). Resistance evolution to Bt maize in the fall armyworm and consideration on IRM strategy in China. Plant Protec..

[32] Chen X., Head G.P., Price P., Kerns D.L., Rice M.E., Huang F., Gilreath R.T., Yang F. (2019). Fitness costs of Vip3A resistance in *Spodoptera frugiperda* on different hosts. Pest Manag. Sci..

[33] Wang N.-M., Li J.-J., Shang Z.-Y., Yu Q.-T., Xue C.-B. (2020). Increased Responses of Phenoloxidase in Chlorantraniliprole Resistance of Plutella xylostella (Lepidoptera: Plutellidae). J. Insect Sci..

[34] Cerenius L., Lee B.L., Söderhäll K. (2008). The proPO-system: Pros and cons for its role in invertebrate immunity. Trends Immunol..

[35] Marinotti O., Ngo T., Kojin B.B., Chou S.P., Nguyen B., Juhn J., Carballar-Lejarazú R., Marinotti P.N., Jiang X., Walter M.F. (2014). Integrated proteomic and transcriptomic analysis of the Aedes aegypti egg shell. BMC Dev. Biol..

[36] Eychenne M., Girard P.-A., Frayssinet M., Lan L., Pagès S., Duvic B., Nègre N. (2022). Mutagenesis of both prophenoloxidases in the fall armyworm induces major defects in metamorphosis. J. Insect Physiol..

[37] Binggeli O., Neyen C., Poidevin M., Lemaitre B. (2014). Prophenoloxidase activation is required for survival to microbial infections in Drosophila. PLoS Pathog..

[38] Karlsson C., Korayem A.M., Scherfer C., Loseva O., Dushay M.S., Theopold U. (2004). Proteomic analysis of the Drosophila larval hemolymph clot. J. Biol. Chem..

[39] Lai S.C., Chen C.C., Hou R.F. (2002). Immunolocalization of prophenoloxidase in the process of wound healing in the Mosquito Armigeres subalbatus (Diptera: Culicidae). J. Med. Entomol..

[40] Snežana M.O., Tatjana V.Č., Jelena S.P., Elvira L.V., Danijela K.K. (2022). Acute toxicity of sublethal concentrations of thiacloprid and clothianidin to immune response and oxidative status of honey bees. Apidologie.

[41] Giglio A., Cavaliere F., Giulianini P.G., Kurtz J., Vommaro M.L., Brandmayr P. (2019). Continuous Agrochemical Treatments in Agroecosystems Can Modify the Effects of Pendimethalin-Based Herbicide Exposure on Immunocompetence of a Beneficial Ground Beetle. Diversity.

[42] Hwang J., Kim Y. (2011). RNA interference of an antimicrobial peptide, gloverin, of the beet armyworm, Spodoptera exigua, enhances susceptibility to Bacillus thuringiensis. J. Invertebr. Pathol..

[43] Schmidt S.L., Christensen B.M. (2003). Hemocyte-mediated phagocytosis and melanization in the mosquito Armigeres subalbatus following immune challenge by bacteria. Cell Tissue Res..

[44] Hillyer J.F., Schmidt S.L., Christensen B.M. (2004). The antibacterial innate immune response by the mosquito Aedes aegypti is mediated by hemocytes and independent of Gram type and pathogenicity. Microbes Infect..

[45] Feng F., Wang P., Zhang Y., An Q., Lin Y., Tong W., Chu P.K., Liang M. (2021). Natural Nanominerals Show Enzyme-Like Activities. J. Nanomater..

[46] Zhang H.-H., Luo M.-J., Zhang Q.-W., Cai P.-M., Idrees A., Ji Q.-E., Yang J.-Q., Chen J.-H. (2018). Molecular characterization of prophenoloxidase-1 (PPO1) and the inhibitory effect of kojic acid on phenoloxidase (PO) activity and on the development of *Zeugodacus tau* (Walker) (Diptera: Tephritidae). Bull. Èntomol. Res..

[47] Karimzadeh J., Wright D.J. (2008). Bottom-up cascading effects in a tritrophic system: Interactions between plant quality and host-parasitoid immune responses. Ecol. Èntomol..

