# Supplementary material for: RNA Interference of Phenoloxidases of the Fall Armyworm, Spodoptera frugiperda, Enhance Susceptibility to Bacillus thuringiensis Protein Vip3Aa19
Source: Insects. 2022 Nov 10;13(11):1041. doi: 10.3390/insects13111041 (PMC9699050; doi:10.3390/insects13111041)
Supplement: Supplementary file 1 [file insects-13-01041-s001.zip › insects-1996846-supplementary.pdf]

Supplementary material

Table S1. Susceptibility of *S. frugiperda* neonates and third instar larvae to Vip3Aa19 toxin.

Different Stages	n	LC_{50} (95% FL) μg/g	Slope ± SE	χ^2	df
1st instar	504	2.11 (1.65–2.60)	2.15±0.27	10.72	19
3rd instar	408	4.98 (4.04–6.23)	2.34±0.20	22.99	15

n, Number of larvae tested. SE, Standard error. 95%FL, 95% fiducial limits.
